# Detection of Carcinoma-Associated Fibroblasts Derived from Mesothelial Cells via Mesothelial-to-Mesenchymal Transition in Primary Ovarian Carcinomas

**DOI:** 10.3390/cancers16152697

**Published:** 2024-07-29

**Authors:** Henar Tomero-Sanz, José Antonio Jiménez-Heffernan, María Concepción Fernández-Chacón, Ignacio Cristóbal-García, Ricardo Sainz de la Cuesta, Lucía González-Cortijo, Manuel López-Cabrera, Pilar Sandoval

**Affiliations:** 1 Tissue and Organ Homeostasis Program, Centro de Biología Molecular Severo Ochoa (CBM), CSIC-UAM, 28049 Madrid, Spain; htomero@cbm.csic.es; 2 Department of Pathology, Hospital Universitario La Princesa, 28006 Madrid, Spain; joseantonio.jimenez@uam.es; 3 Hospital Universitario de la Zarzuela, 28023 Madrid, Spain; mcfernandezch@sanitas.es (M.C.F.-C.); icristobal@sanitas.es (I.C.-G.); 4 Hospital Universitario QuirónSalud Madrid, 28223 Madrid, Spain; ricardo.sainz@quironsalud.es (R.S.d.l.C.); lucia.gonzalezc@quironsalud.es (L.G.-C.); 5 Department of de Medicine, Facultad de Biomédica y Ciencias de la Salud, Universidad Europea de Madrid, 28670 Madrid, Spain

**Keywords:** ovarian cancer, primary tumor, mesothelial cells, carcinoma-associated fibroblasts, mesothelial-to-mesenchymal transition

## Abstract

**Simple Summary:**

Approximately 70–80% of patients with epithelial ovarian cancer (OC) experience peritoneal metastasis. Identification of mesothelial-to-mesenchymal transition (MMT) in the peritoneum may guide the accurate treatment and follow-up of patients with advanced OC. The aim of this study was to evaluate whether MMT took place in primary OC biopsies. We suggest the identified mesothelial-derived carcinoma-associated fibroblasts in primary tumors could impact the clinical progression of patients with OC.

**Abstract:**

Carcinoma-associated fibroblasts (CAFs) are highly accumulated in the tumor-surrounding stroma of primary epithelial ovarian cancer (OC). CAFs exert important functions for the vascularization, growth, and progression of OC cells. However, the origin of CAFs in primary OC had not yet been studied, and they were assumed to arise from the activation of resident fibroblasts. Here, we compared CAFs in the ovary to CAFs found in peritoneal metastases from patients with advanced OC. Our findings show that CAFs from primary tumors and peritoneal metastases share the expression of mesothelial markers. Therefore, similar to peritoneal carcinomatosis, CAFs in primary ovarian carcinomas may originate from mesothelial cells via a mesothelial-to-mesenchymal transition. The detection of mesothelial-derived CAFs in tumors confined to the ovary and identification of biomarkers could be the key to the early detection of OC and peritoneal spread.

## 1. Introduction

The ovarian surface epithelium (OSE) is a single continuous layer of flat-to-cuboidal cells loosely attached to the basement membrane that covers the ovaries. The cells that form the OSE are sometimes referred to as mesothelial-like, since they share a common embryological background and characteristics with peritoneal mesothelial cells (MCs) [1]. The OSE directly develops from the coelomic epithelium. The Müllerian duct, an embryological precursor of the female reproductive organs, including the fallopian tubes, uterus, cervix, and upper vagina, also derives from this coelomic layer, which has a mesodermal origin [2,3,4]. In fact, the OSE is frequently described in the literature as the ovarian mesothelium [3]. On the other hand, the OSE is continued by the mesovarium—a flat sheet of peritoneum associated with the ovaries—which projects from the posterior surface of the broad ligament of the uterus and attaches to the hilum of the ovaries, enclosing their neurovascular supply, and forming the mesentery of the ovaries [1,3,5,6]. Approximately 90% of ovarian cancer (OC) arises from the OSE, while a small portion originates from germ cells or sex-cord stromal tissues. Epithelial OC is classified into five sub-groups: low-grade serous ovarian cancer (LGSOC), high-grade serous ovarian cancer (HGSOC), mucinous, endometroid, and clear cell ovarian carcinomas [7]. HGSOC is the most common OC subtype, accounting for about 70% of OC; it is also the most aggressive and lethal subtype [5,8]. HGSOC is thought to arise from either the transformation of the OSE or precursor lesions in the fallopian tubal epithelium [1,4,7].

**Figure 1:**
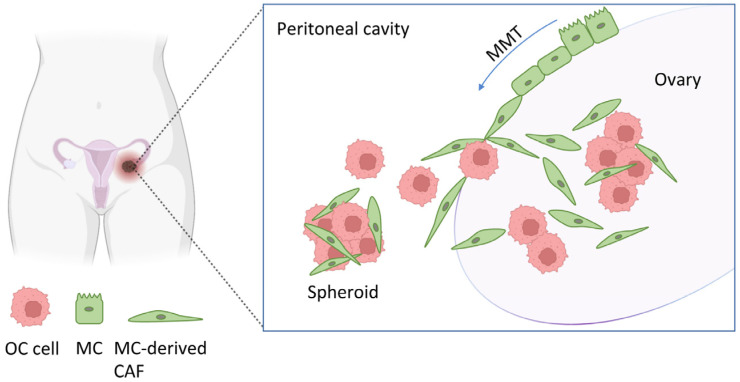
Mesothelial-to-mesenchymal transition in the ovary. During ovarian cancer (OC) tumorigenesis, mesothelial cells (MCs) that line the ovary can undergo mesothelial-to-mesenchymal transition (MMT) and lead to MC-derived carcinoma-associated fibroblasts (CAFs). These CAFs can invade the submesothelial area and provide a suitable microenvironment for OC progression. Spheroids containing OC cells and CAFs detach from the primary tumor and disseminate through the peritoneal cavity.

Primary ovarian/fallopian tube carcinoma frequently metastasizes to the peritoneum. The peritoneum is composed of a monolayer of MCs resting on connective tissue with few fibroblasts, immune cells, adipocytes, and vessels [9]. It includes the parietal peritoneum, which covers the internal wall of the abdominal cavity, and the visceral peritoneum, which lines the abdominal organs. Both serosa monolayers share mesothelial markers such as cytokeratins, calretinin, mesothelin, and Wilms tumor 1 (WT1) [10]. In ovarian carcinomatosis, cancer cells detach from the primary tumor, disseminate through the peritoneal cavity, and attach to and invade through the mesothelium (Figure 1). Our group was the first to describe that a sizeable population of carcinoma-associated fibroblasts (CAFs) derived from MCs through a mesothelial-to-mesenchymal transition (MMT) and accumulated in the peritoneal stroma, promoting OC adhesion to and invasion through the peritoneum, vascularization, and proliferation of OC cells. Thus, MMT is a key event in the pathogenesis of ovarian carcinomatosis [11,12,13,14,15,16]. During MMT, which is an epithelial-to-mesenchymal transition (EMT)-like process, MCs lose apico-basolateral polarity and intercellular adhesions, changing from a cobblestone-like morphology to a fibroblast-like one. In this process, MCs also increase their migratory and invasive capacity, invading the submesothelial area, and enhance the production of extracellular matrix (ECM) components [17]. In parallel, an increased expression of metalloproteinases (MMPs) that degrade the basement membrane is observable during MMT. These changes are a result of a profound genetic reprogramming, characterized by the downregulation of epithelial markers [18,19] and the acquisition of α-smooth muscle actin (α-SMA), a marker of myofibroblasts (i.e., activated fibroblasts), among others [20].

CAFs are myofibroblasts that are present in the stroma of solid tumors [21] and can derive from various sources, depending on the cancer type and/or the individual area within the tumor. The activation of resident fibroblasts is considered the main origin of CAFs in the tumor microenvironment [22]. Alternative sources of CAFs include bone marrow-derived precursors, endothelial cells through an endothelial-to-mesenchymal transition (EndTM), adipocytes [23], and epithelial cells through EMT [13,24,25,26].

Here, we aimed to compare CAFs from primary ovarian carcinomas to CAFs from peritoneal carcinoma implants. Our results show that CAFs in the ovary express mesothelial markers in a similar manner to CAFs derived from MCs through MMT in the peritoneal metastases of OC.

## 2. Materials and Methods

### 2.1. Patient Samples and Cell Cultures

Human peritoneal MCs (HPMCs) obtained from omentum samples of patients undergoing unrelated abdominal surgery were used as controls. The samples were trypsinized (0.25% trypsin solution containing 0.02% EDTA), while occasionally agitated, for 15 min at 37 °C. Cells were cultured in Earle’s M199 medium (Biological Industries, Beit Haemek, Israel) supplemented with 20% fetal bovine serum (FBS) and 2% Biogro-2 (Biological Industries, Beit Haemek, Israel). As a positive control of MMT, 0.5 ng/mL transforming growth factor-β1 (TGF-β1; R&D Systems, Minneapolis, MN, USA) plus 2.5 ng/mL interleukin-1β (IL-1β; R&D Systems) (T + I) were added to HPMCs for 72 h, which has been previously described as an effective way to induce MMT in vitro [27,28,29].

CAFs for ex vivo expansion were obtained from fresh tumor tissue samples of patients with HGSOC undergoing cytoreductive surgery. Patients did not receive chemotherapy before surgery. Ovarian carcinomas and matched secondary tumors from three patients (Supplementary Table S1) were cut into small fragments of approximately 3 mm in diameter and carefully placed on high binding culture plates (25 cm^2^ flask; Corning, New York, NY, USA) [30]. CAFs were allowed to grow in Earle’s M199 medium (Biological Industries) supplemented with 20% of FBS (Thermo Fisher Scientific, Waltham, MA, USA) and 2% Biogro-2 (Biological Industries) at 37 °C and 5% CO_2_ until they reached 75% of confluence. The remaining tissue fragments were removed, and cells remained stable for at least 2 passages before use.

Brightfield images of cell cultures were obtained with 10× objective using a Nikon COOLPIX 4500 (Tokyo, Japan) camera coupled to a Nikon Eclipse TS100 microscope (Tokyo, Japan).

Biopsies from primary ovarian tumors and their corresponding peritoneal metastases from a total of nine patients with HGSOC were used for immunohistochemical analysis. Patient information has been summarized in Supplementary Table S2.

This study was carried out in accordance with Good Clinical Practice guidelines and applicable regulations, as well as the ethical principles that have their origin in the Declaration of Helsinki. Informed written consent was obtained from the patients, with the approval of the Clinical Ethics Committee of Puerta de Hierro Hospital and Majadahonda Hospital (ethics approval number: 11.17; Madrid, Spain) and Fundación Jiménez Díaz (ethics approval number: 11/17; Madrid, Spain).

### 2.2. Immunofluorescence

Immunofluorescence staining was performed to visualize α-SMA (Clone 1A4, 1:3000, Sigma-Aldrich, St. Louis, MI, USA) in cell cultures. Cells were plated on 22 mm^2^ coverslips placed in 24-well tissue culture plates and fixed in 4% paraformaldehyde. Samples were then permeabilized in 0.1% NP-40 and blocked for non-specific unions in 0.1% bovine serum albumin, 0.2% NP40, and 0.05% Tween 20 diluted in PBS 1x. After incubating the primary antibody, samples were incubated with a secondary anti-mouse antibody conjugated to Alexa Fluor-488 (Thermo Scientific, Waltham, MA, USA). Finally, nuclei were stained with 4,6-diamidino-2-phenylindole (DAPI; Merck Millipore, Burlington, MA, USA). Confocal images were captured with an LSM710 confocal microscope (Zeiss, Oberkochen, Germany).

### 2.3. Reverse Transcription—Quantitative PCR (RT-qPCR)

mRNAs of MMT genes were analyzed by RT-qPCR in cell cultures of HPMCs and in matched primary and secondary tumors. Cells were lysed in TRIzol Reagent (Ambion, Van Allen Way, Carlsbad CA, USA) and manufacturer’s instructions were followed to extract total RNA. An amount of 2 μg RNA was used to obtain complementary DNA by reverse transcription (Applied Biosystems, Cheshire, UK), prior to performing quantitative PCR in a LightCycler 480 II (LightCycler 480 1.5.0 software; Roche Diagnostics, Basel, Switzerland), using an SYBR Green kit (Roche Diagnostics). Specific primers for E-cadherin, calretinin, vascular endothelial growth factor receptor 2 (KDR/VEGFR-2), collagen I, TGF-β1, vascular endothelial growth factor A (VEGF-A), neuropilin 1 (NRP-1), and histone H3 are shown in Supplementary Table S3. Samples were normalized to histone H3 values.

### 2.4. Immunoblotting

HPMCs, HPMCs treated with TGF-β1 plus IL-1β (T + I), and CAFs from primary and secondary tumors were lysed using RIPA buffer with protease and phosphatase inhibitor cocktail (Thermo Scientific, USA). Total protein concentration was determined using Pierce^®^ BCA Protein Assay Kit (Thermo Scientific), following the manufacturer’s indications. An equal amount of protein (30 μg) was diluted in Laemmli buffer and resolved by SDS-PAGE (7% acrylamide). Proteins were transferred to nitrocellulose membranes, 0.45 μm (Bio-Rad, Hercules, CA, USA), and incubated with PDPN (Origene Technologies, Rockville, MD, USA), pan-CK (Sigma Aldrich), and α-tubulin (Sigma Aldrich) antibodies. Signals were detected by chemiluminescence with SuperSignal^®^ West Pico Chemiluminiscent Substrate (Thermo Scientific, Massachusetts, MA, USA), using Amersham Imager 680 system (GE Healthcare, Illinois, IL, USA). Densiometric analysis was performed using ImageJ 1.52k.

### 2.5. Immunohistochemistry

For immunohistochemical analysis, patient biopsies were fixed in neutral-buffered 3.7% formalin and embedded in paraffin to obtain serial 3 μm sections. Deparaffinized tissues were heated to expose the hidden antigens using Real Target Retrieval Solution containing citrate buffer, pH 6.0 (Sigma-Aldrich, USA). Endogenous peroxidase was blocked with Real Peroxidase-Blocking Solution (Dako, Glostrup, Denmark). The following primary antibodies were tested: podoplanin (PDPN) (clone NZ-1, 1:500, Origine, Rockville, MD, USA), pan-cytokeratin (pan-CK, clone PCK-26; Sigma-Aldrich), calretinin (clone DC8, Invitrogen by Life Technologies, Carlsbad, CA, USA), α-SMA (clone 1A4; Sigma-Aldrich), and fibroblast activation protein (FAP, clone EPR20021; Abcam, Cambridge, UK). Biotinylated goat anti-rat IgG, anti-mouse IgG, or anti-rabbit IgG (Vector Laboratories, Carlsbad, CA, USA) were applied to detect primary antibodies. Complexes were visualized using the VECTASTAIN Elite ABC Reagent, Peroxidase, R.T.U. (Vector Laboratories) and 3,3’-diaminobenzidine (DAB; Dako) as chromogen. Tissue sections were counterstained with hematoxylin.

### 2.6. Statistical Analysis

Statistical analyses were performed using GraphPad Prism version 8.4.0 (455) for macOS (GraphPad software, version 8.4.0.(455), San Diego, California, CA, USA). Results were represented as mean ± standard error of the mean (SEM) in bar graphics. Data groups were compared with the non-parametric Mann–Whitney rank sum U-test. A *p*-value < 0.05 was considered statistically significant.

## 3. Results

### 3.1. Mesothelial-to-Mesenchymal Transition Detected in CAFs Isolated from Primary Ovarian Tumors

**Figure 2:**
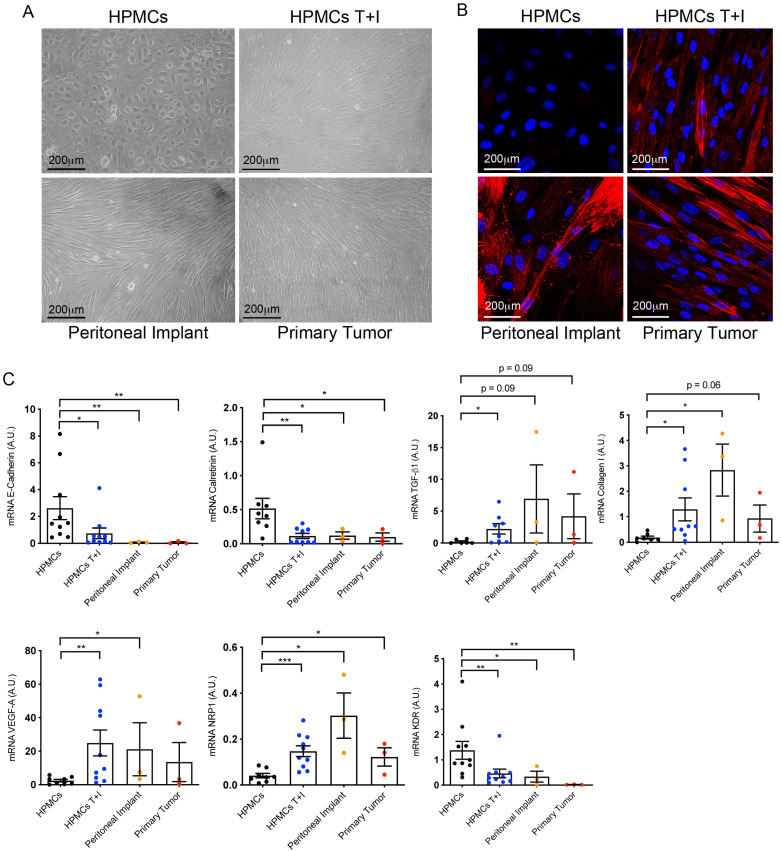
Mesothelial-to-mesenchymal transition in CAFs isolated from primary ovarian tumors. (**A**) Representative microscopy images of control HPMCs, HPMCs treated with TGF-β1 plus IL-1β (T + I), cells with a spindle-like morphology from peritoneal implants and from primary tumors in culture. (**B**) Representative immunofluorescence images of α-SMA expression in HPMCs, HPMCs treated with TGF-β1 plus IL-1β (T + I), CAFs from a peritoneal implant and primary tumor. (**C**) qRT-PCR analysis of MMT markers in HMCs, HPMCs T + I, CAFs from peritoneal implants and from ovarian carcinomas of the same patients. Bar graphs represent mean ± SEM; symbols represent statistical differences between groups (* *p* ≤ 0.05; ** *p* ≤ 0.001; *** *p* ≤ 0.0001).

Cells from primary OC tumors and peritoneal metastases presented a spindle-like morphology, similar to that of HPMCs that had transdifferentiated by undergoing an in vitro MMT induced with T + I; this appearance differed from that of control HPMCs, which maintained a cobblestone morphology (Figure 2A). The transdifferentiation of HPMCs to myofibroblasts was validated by staining cultures with α-SMA. Transdifferentiated cells isolated from primary OC tumors and their corresponding peritoneal metastases expressed α-SMA in a similar manner to HPMCs transdifferentiated in vitro. HPMCs did not express α-SMA (Figure 2B).

At a molecular level (Figure 2C), the expression of E-cadherin, which is an EMT hallmark, was strongly repressed in cells isolated from primary and secondary tumors, compared to HPMCs. Likewise, calretinin, a recognized mesothelial marker, had a significantly decreased expression in cells isolated from both primary and secondary tumors. A decrease in mesothelial markers, such as PDPN and cytokeratins, was determined in the CAFs of primary and secondary tumors by immunoblot assay (Supplementary Figures S1 and S2). Conversely, the CAFs of primary tumors showed a significant upregulation of MMT-associated markers including TGF-β1 and the ECM component collagen I in a similar manner to HPMCs transdifferentiated in vitro. Molecular analysis showed a trend towards the increased expression of the angiogenic factor VEGF-A in the CAFs of primary carcinomas compared to HPMCs, although statistical significance was not reached. Decreased expression of the VEGF-A receptor KDR/VEGFR-2, involved in proliferation, and increased expression of the VEGF-A co-receptor NRP-1, involved in invasion, were observed in the CAFs of primary and secondary tumors compared with HPMCs. Both dysregulations were similar to those observed in HPMCs transdifferentiated in vitro and CAFs from peritoneal metastases (Figure 2C). Taken together, these results suggest that CAFs from primary and secondary tumors have undergone an MMT.

### 3.2. CAFs Expressing Mesothelial Cell Markers Are Present in Primary Ovarian Carcinomas

**Figure 3:**
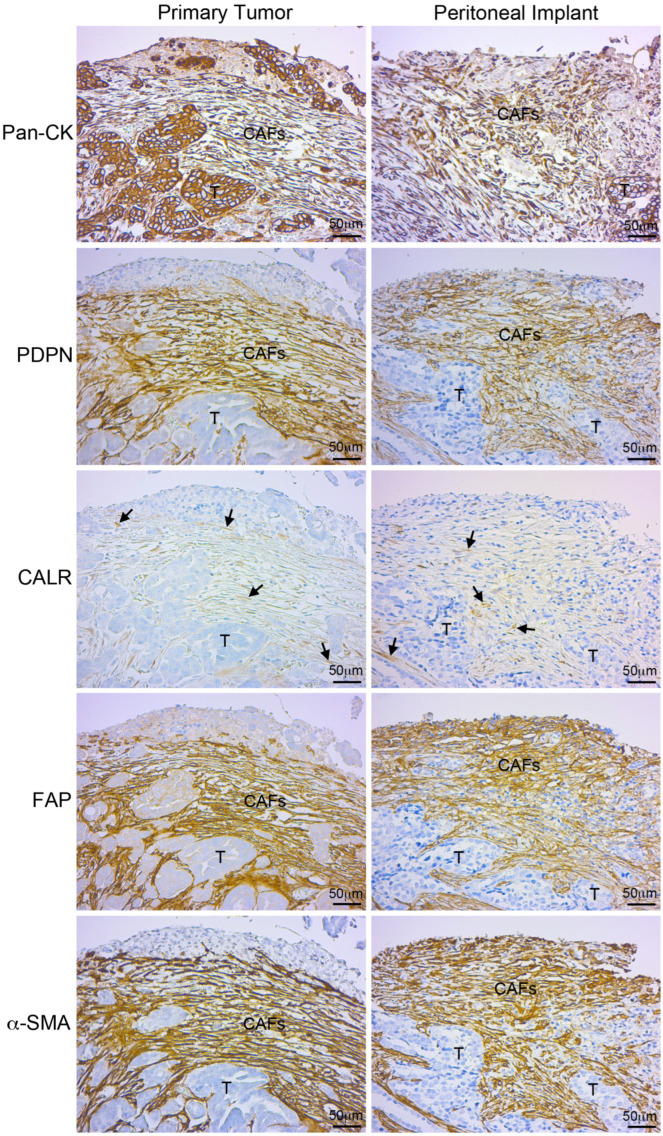
CAFs express mesothelial cell markers in primary ovarian carcinomas. Immunohistochemical analysis revealed co-localization of mesothelial (pan-CK, PDPN, and calretinin [CALR]) and CAF (FAP and α-SMA) markers in the ovaries and in peritoneal metastases. Arrows point to CAFs. Scale bars = 50 μm. CAFs: Carcinoma-associated fibroblasts; T: tumor.

Immunohistochemical staining of serial tumor sections revealed the expression of mesothelial markers (PDPN and calretinin) in CAFs located in areas surrounding tumor cells in primary ovarian tumors and secondary peritoneal metastases (Figure 3). A low staining intensity was observed for calretinin in the stroma of primary tumors and peritoneal metastases. This result is consistent with the low quantification of calretinin at the mRNA level (Figure 2C). Positive pan-CK staining was detected in both the tumor cells and CAFs. The localization of the positive staining of mesothelial markers and CAFs (FAP and α-SMA) was very similar in the primary and secondary tumors (Figure 3).

## 4. Discussion

Ovarian carcinomatosis is a complex metastatic process involving tumor cells and a supporting stroma formed by a fibrotic, immune, and microvascular microenvironment. In the context of OC metastasis, CAFs of mesothelial origin acquire a notorious relevance, since they constitute an important population in the peritoneal environment and play a significant role in OC progression [30]. MMT has been previously described in peritoneal ascitic fluid as well as in peritoneal biopsies from patients with advanced OC [13,31,32]. However, the study of the origin of CAFs surrounding the tumor at its primary location (ovary) had been neglected.

Here, we analyzed the expression of MMT markers in ex vivo-expanded cells from primary ovarian tumors and compared them with cells from peritoneal tumor explants. The fibroblast-like morphology and expression of α-SMA indicated that cultured cells from primary and secondary tumors were myofibroblasts. In cells isolated from primary tumors, we observed the downregulation of mesothelial markers, such as E-cadherin and calretinin, and the overexpression of mesenchymal markers, including TGF-β1 and collagen I, suggesting they underwent MMT similar to that observed in MCs stimulated in vitro or in CAFs expanded ex vivo from peritoneal metastases. We also found an upregulation of VEGF-A and the co-receptor NRP-1, as well as an underexpression of the receptor KDR/VEGFR-2 in the CAFs of primary and secondary tumors compared to the control MCs. On this note, previous studies from our group pointed to mesothelial-derived CAFs as the main producers of VEGF in the peritoneal metastatic niche [13,31]. The functional relevance of the switch of the KDR/VEGFR-2 and NRP-1 during MMT has also previously been characterized. In fact, treating MCs with neutralizing anti-VEGF or anti-NRP-1 antibodies showed that both molecules played a relevant role driving MCs from a proliferative response to invading the peritoneal membrane [33].

Moreover, we analyzed the biopsies of primary ovarian carcinomas to determine whether CAFs expressed mesothelial markers. PDPN was intensely stained in FAP/α-SMA double-positive areas in serial sections from primary and secondary tumors. PDPN expression in physiological conditions is limited to the mesothelium that lines serous cavities and to the endothelium of lymphatic vessels [34,35,36,37]. Interestingly, PDPN-positive CAFs have been shown to predict poor cancer prognosis [38]. In this regard, we recently showed that the immunohistochemical detection of CAFs expressing PDPN was mainly associated with peritoneal metastases of aggressive histological subtypes of epithelial OC [32]. Taken together, these data suggest intratumoral PDPN-positive CAFs could also have a mesothelial origin in primitive neoplasms.

Calretinin is a calcium adhesion protein that in healthy conditions is expressed mainly in the nervous system and MCs. In disease, calretinin is expressed in MC neoplasms (mesothelioma) [39,40]. However, in peritoneal metastases, calretinin expression is limited to the surrounding stroma of epithelial tumor cells [13,31,41]. Consistent with the transcriptional data in the ex vivo cultures described above, calretinin weakly labeled stromal zones that were triple positive for PDPN, FAP, and α-SMA in ovarian biopsies. This weak staining could be related to a terminal time point of the MMT, when mesothelial markers are close to cease expressing.

Here, we used PDPN and calretinin as the main mesothelial markers. Our data suggest that CAFs accompanying primary ovarian carcinomas and CAFs surrounding peritoneal metastases have a mesothelial background. The detection of PDPN and calretinin in intraovarian CAFs suggests that they could derive from the adjacent mesovarium through MMT. Of note, PDPN and calretinin staining is limited to CAFs and are not expressed in OC cells. In line with these results, our group previously demonstrated that CAFs can derive from the MCs that line the visceral peritoneum through MMT in locally advanced primary colorectal carcinomas; this was determined by the submesothelial detection of mesothelial markers, including calretinin, mesothelin, and cytokeratin 7 [10]. However, in the context of OC, PDPN and calretinin are also detectable in the OSE, as well as in cortical inclusion cysts (formed by invaginations of OSE) [42]. Therefore, the close similarity of both ovarian-associated surfaces complicates identifying with precision the real origin of CAFs in primary OC. The mesenchymal conversion via EMT of the epithelial cells that cover the ovarian surface is a possibility that cannot be discarded. In fact, the mesothelium of continuity and the OSE have in common the basal expression of several markers, including E-cadherin, N-cadherin, cytokeratins, mesothelin, WT1, CA125/MUC16, and NRP-1 [5,42]. Interestingly, among these mesothelial/epithelial ovarian surface markers, CA125, N-cadherin, cytokeratins, mesothelin, and NRP-1 are also frequently detected in epithelial OC cells [43,44,45,46,47]. In fact, ovarian stem cells in the OSE may be responsible for epithelial ovarian tumorigenesis via EMT [1,6]. On the other hand, primary ovarian mesothelioma is rare. This evidence would preferentially support an epithelial rather than a mesothelial origin for CAFs [48]. Further studies using “omic” technologies that compare the expression profiles of CAFs from primary tumors and CAFs from metastatic sites could help clarify the similarities and/or differences between CAFs from distinct serous locations. In addition, the characterization of the molecular profile of intraovarian CAFs could be relevant to predict OC progression and/or identify new potential therapeutic targets [43]. In this regard, Gao et al. described the presence of CAFs in peritoneal spheroids from patients with HGSOC, but not LGSOC [49]. Accordingly, ascitic fluid-isolated CAFs from HGSOC tumors presented a more robust MMT signature than LGSOC tumors [32]. Therefore, the detection of MC-derived CAFs in primary tumor biopsies could be of interest to classify patients with stage I OC according to the aggressiveness of the tumor. Nowadays, women of childbearing age with OC may be subjected to a fertility-sparing surgery; on this note, detection of MMT in the primary tumor could help guide the use of a radical vs. a conservative surgery. Future studies should aim to identify MMT biomarkers that could be used to predict OC progression using non-invasive techniques (evaluation of serum, peritoneal washing, or ascitic fluid). Additionally, the identified MC-derived CAFs in primary ovarian tumors could provide insights for the design of new therapeutic options. OC patients at early stages of tumor progression might benefit from MMT-targeted antibody–drug conjugates (ADCs). In this regard, we recently demonstrated that an anti-FAP ADC interferes with peritoneal metastasis in a mouse model of ovarian carcinomatosis [32].

One limitation of our study is the small patient cohort. Further studies with a large series of patients with different OC histological subtypes could draw more robust conclusions to support the findings of our study. The lack of mesothelial-specific markers limits the study of MC-derived CAFs in primary tumors, which could be formally demonstrated by lineage-tracing studies in animal models.

## 5. Conclusions

This study shows, for the first time, the presence of CAFs originating from MCs through MMT in primary ovarian tumors. The identification of the presence of MMT-derived CAFs in the ovary via non-invasive approaches could be of value in the early detection of OC. Characterization of these CAFs may help design therapeutic strategies to target them, as CAFs play major roles in the progression of OC. MMT-targeted treatments could delay or decelerate peritoneal metastasis, ultimately benefiting patients with OC.

## Data Availability

The original contributions presented in the study are included in the article/Supplementary Material, further inquiries can be directed to the corresponding authors.

## Supplementary Materials

The following supporting information can be downloaded at: https://www.mdpi.com/article/10.3390/cancers16152697/s1, Figure S1: Decreased expression of PDPN and pan-cytokeratin in CAFs isolated from primary and secondary tumors; Figure S2. Original western blot membranes corresponding to Supplementary Figure S1; Table S1: patients considered for ex vivo studies; Table S2: patients considered for immunohistochemical studies; Table S3: specific primers for real-time PCR.
