# Supplementary material for: Detection of Carcinoma-Associated Fibroblasts Derived from Mesothelial Cells via Mesothelial-to-Mesenchymal Transition in Primary Ovarian Carcinomas
Source: Cancers (Basel). 2024 Jul 29;16(15):2697. doi: 10.3390/cancers16152697 (PMC11311419; doi:10.3390/cancers16152697)
Supplement: Supplementary file 1 [file cancers-16-02697-s001.zip › Supplementary Figures.pdf]

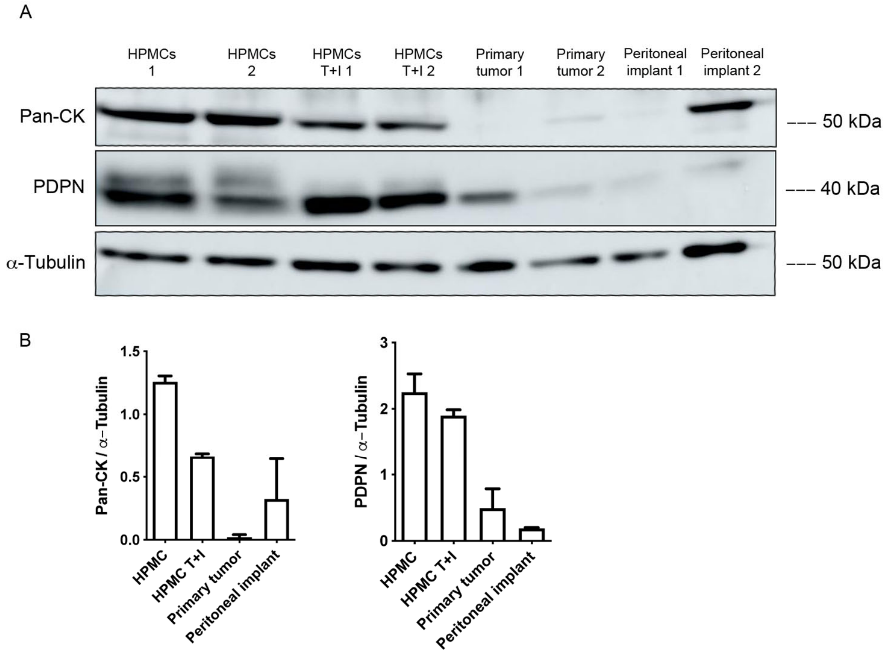

Supplementary figure S1. **Decreased expression of PDPN and pan-cytokeratin in CAFs isolated from primary and secondary tumors.** (A) Western blot image of HPMCs (n=2), HPMCs treated with TGF-β1 plus IL-1β (T+I) (n=2) and CAFs isolated from 2 independent primary tumor and peritoneal implant tissue samples. (B) Western blot quantification normalized with respect to the signal obtained for α-tubulin.

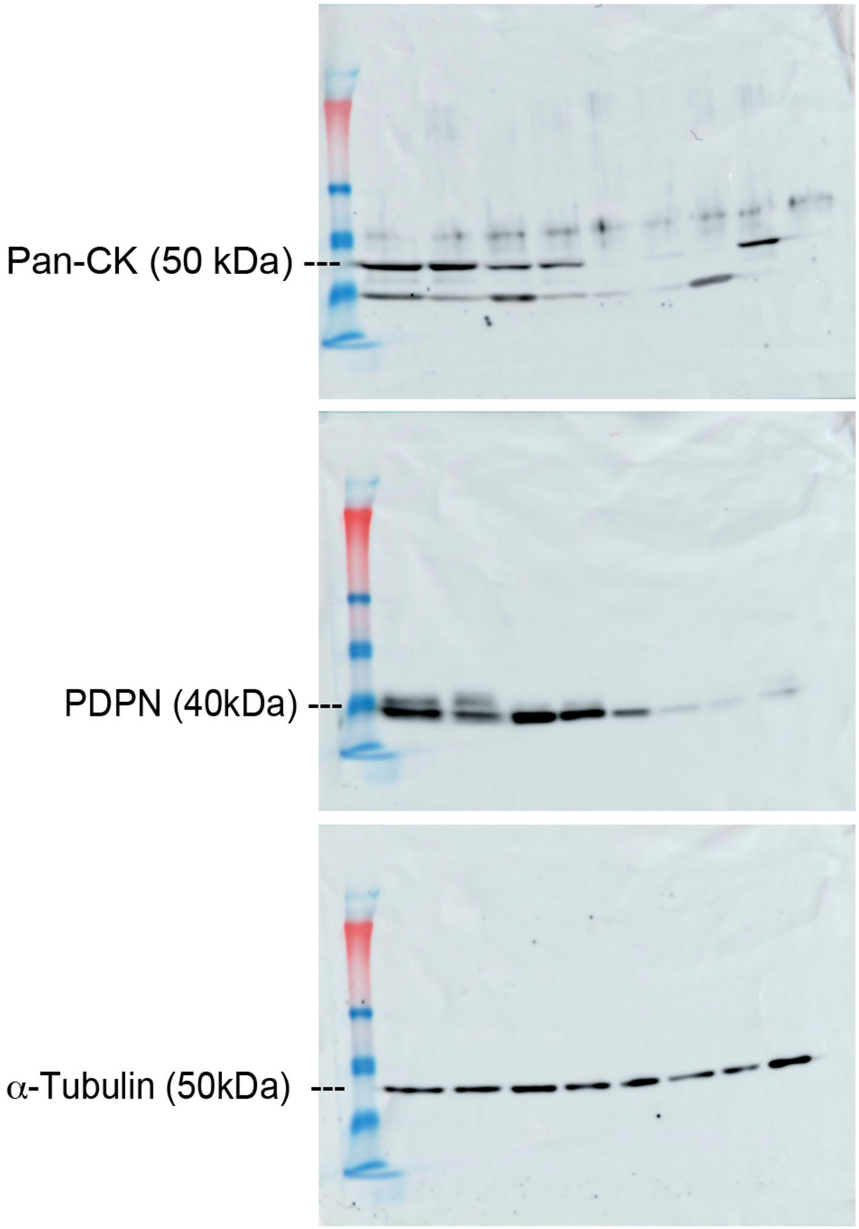

Supplementary figure S2. Original western blot membranes corresponding to Supplementary figure S1.
