# Supplementary material for: Detection of Carcinoma-Associated Fibroblasts Derived from Mesothelial Cells via Mesothelial-to-Mesenchymal Transition in Primary Ovarian Carcinomas
Source: Cancers (Basel). 2024 Jul 29;16(15):2697. doi: 10.3390/cancers16152697 (PMC11311419; doi:10.3390/cancers16152697)
Supplement: Supplementary file 1 [file cancers-16-02697-s001.zip › Supplementary Tables.pdf]

Supplementary Table S1: patients considered for *ex vivo* studies

Patient ID	Age	Ethnicity	OC subtype	Treatment other than cytoreductive surgery	Fresh sample availability	
					Primary tumor	Secondary tumor
#1	71	White/Caucasian	HGSOC	No	Yes	Yes
#2	72	White/Caucasian	HGSOC	No	Yes	Yes
#3	71	White/Caucasian	HGSOC	No	Yes	Yes

Supplementary Table S2: patients considered for immunohistochemical studies

Patient ID	Age	Ethnicity	OC subtype	Treatment other than cytoreductive surgery	Formalin-fixed paraffin-embedded (FFPE) biopsy availability	
					Primary tumor	Secondary tumor
#4	74	White/Caucasian	HGSOC	No	Yes	Yes
#5	64	White/Caucasian	HGSOC	No	Yes	Yes
#6	48	White/Caucasian	HGSOC	No	Yes	Yes
#7	54	White/Caucasian	HGSOC	No	Yes	Yes
#8	71	White/Caucasian	HGSOC	No	Yes	Yes
#9	67	White/Caucasian	HGSOC	No	Yes	Yes
#10	48	White/Caucasian	HGSOC	No	Yes	Yes
#11	77	White/Caucasian	HGSOC	No	Yes	Yes
#12	48	White/Caucasian	HGSOC	No	Yes	Yes

Supplementary Table S3: specific primers for real-time PCR

Gene	Forward primer	Reverse primer	*Tm* (ºC)
E-cadherin	5'-TGAAGGTGACAGAGCCTCTG-3'	5'-TGGGTGAATTCGGGCTTGTT-3'	62
Calretinin	5'-ACTTTGACGCAGACGGAAATG-3'	5'-GAAGTTCTCTTCGGTTGGCAG-3'	62
KDR / VEGFR2	5'-TGAGCATGGAAGAGGATTCTG-3'	5'-CTCTTTCGCTTACTGTTCTGC-3'	62
Collagen I	5'-GCTATGATGAGAAATCAACCG-3'	5'-GCTTCCCCATCATCTCCATTC-3'	64
TGF-β1	5'-TGAACCGGCCTTTCCTGCTTCTCATG-3'	5'-CGGAAGTCAATGTACAGCTGCCGC-3'	70
VEGF-A	5'-GCAGAAGGAGGAGGGCAGAAT-3'	5'-TATGTGCTGGCCTTGGTGAGG-3'	60
NRP1	5'-AAGGTTTCTCAGCAAACTACAGTG-3'	5'-GGGAAGAAGCTGTGATCTGGTC-3'	68
Histone H3	5'-AAAGCCGCTCGCAAGAGTGCG-3'	5'-ACTTGCCTCCTGCAAAGCAC-3'	62
